# Outcomes Following Achilles Tendon Ruptures in the National Hockey League: A Retrospective Sports Database Study

**DOI:** 10.3390/jcm14155471

**Published:** 2025-08-04

**Authors:** Bradley A. Lezak, James J. Butler, Rohan Phadke, Nathaniel P. Mercer, Sebastian Krebsbach, Theodor Di Pauli von Treuheim, Alexander Tham, Andrew J. Rosenbaum, John G. Kennedy

**Affiliations:** 1 Foot and Ankle Division, Department of Orthopaedic Surgery, NYU Langone Health, New York City, NY 10002, USA; 2 Albany Medical Center, Albany, NY 12208, USA

**Keywords:** Achilles tendon rupture, return to play, performance metrics, ice hockey

## Abstract

**Background:** The purpose of this study was to evaluate Achilles tendon ruptures (ATR) in NHL players and the effects on return to play and player performance metrics. The incidence, mechanism of injury, management strategy, return to play (RTP), and post-injury were assessed from official online sports databases. **Methods**: A retrospective review of NHL players who sustained a partial or complete tear of the Achilles tendon from 2008 to 2024 was performed. Data were collected from NHL injury databases and media reports, and included player demographics, injury mechanism, treatment, and post-injury performance metrics. A Wilcoxon signed rank test was used to compare pre-injury and post-injury performance metrics, with significance set at *p* < 0.05. **Results:** Here, 15 NHL players with a mean age of 27.8 years were identified, with a prevalence rate of 0.125 injuries per 10,000 athletic exposures. Overall, 73.3% of ATRs were non-contact in nature, with 60.0% of ATRs occurring during off-season training. Fourteen players were managed with non-operative treatment, with no re-ruptures reported. The RTP rate was 93.3%, with players missing a mean number of 45.7 games. However, there was a deterioration in post-injury performance metrics, including games played per season, plus/minus rating, and time on ice per game post-injury. **Conclusions**: This study found that Achilles tendon ruptures are an uncommon injury in NHL players, with a prevalence rate of 0.125 injuries per 10,000 athletic exposures. A high RTP rate of 93.3% was observed in this cohort. However, there was a deterioration in post-injury performance metrics, including games played per season, plus/minus rating, and time on ice per game post-injury, highlighting the potential devastating sequelae of ATRs in elite NHL athletes.

## 1. Introduction

Ice hockey is an intense, high-speed sport characterized by frequent collisions and high-impact interactions with other players, equipment, and rink boards. This environment creates a high risk of lower extremity injuries, which comprise 27–50% of all reported injuries in the National Hockey League (NHL) [1]. Foot and ankle injuries experienced by athletes participating in the NHL can be complex and can have substantial implications on the athlete’s ability to return to play (RTP) [2]. These injuries can significantly disrupt performance and impose a high financial cost on teams, resulting in players missing 9.9 games per season, and contributing to a mean loss of USD 283,100 in player salary per injury [3].

The Achilles tendon is a strong fibrous band created by the merging of the gastrocnemius and soleus muscles and attaches to the back of the calcaneus. It is the thickest, strongest, and largest tendon in the human body [4,5] and is the most commonly ruptured tendon in the lower extremity [6,7,8,9]. The Achilles tendon plays a pivotal role in generating plantar flexion of the foot, and is a key movement required for activities such as running, walking, jumping, and ice skating [10,11]. It can bear loads up to seven times the body weight [12] during activities like running, which creates high tensile forces through muscle contractions and ground reaction forces during the push-off phase of gait [12]. Its strength is derived from its gross anatomy as well as the microscopic ultrastructure. The Achilles tendon twists counterclockwise on the right and clockwise on the left as it descends [13], which reflects its function in force transmission from the heads of the gastrocnemius and soleus muscles across a wide range of ankle joint motions. Through this unique anatomy, mechanical forces are effectively distributed, however, subject the tendon core to tensile, compressive, and shear stresses [13]. Kager fat pads and the retrocalcaneal bursa are found between the bone and the tendon at the bony insertion site and reduce friction during movement, as well facilitating mechanical function [14]. The Achilles tendon is comprised primarily of type I collagen fibers organized in parallel. The tenocytes, an extracellular matrix (ECM) producing fibroblast-like cells, are mostly responsible for synthesizing and maintaining the ECM [15]. Collagen type I provides the tensile strength of the tendon, and the tenocytes respond to mechanical loading by increasing ECM turnover [16,17]. The pathophysiology of an Achilles tendon rupture typically involves two key factors: (1) tendinosis, which refers to degenerative changes within the tendon, and (2) an acute eccentric contraction strong enough to tear the tendon [10]. The degenerative changes in tendinosis are a result of the healing process, where collagen type III is haphazardly laid down to patch the damage in the tendon ECM [18]. This creates a biomechanically weaker tendon, due to the relatively lower collagen type I content, and therefore is less resilient to stresses placed upon it [18,19]. The Achilles tendon typically ruptures 2 to 6 cm above the calcaneal insertion, which is the area at which the tendon fibers twist and the blood supply is diminished [13].

While Achilles tendon ruptures commonly occur in recreational athletes in the general population at a rate ranging from 8.3 to 24 per 100,000 individuals [20], collegiate and elite athletes are at risk of sustaining this injury at a higher incidence due to the intense physical demands of professional sports [20,21]. In ice hockey, the stress on the Achilles tendon is multifaceted. Ice skaters have higher lifetime rates of Achilles tendon injuries, with ultrasound changes correlated with repeated tendon loading and the plantarflexed posture in stiff boots [22], thereby contributing to tendinosis. During explosive movements, such as acceleration from a stationary position, or making a sudden stop, the ankle is plantarflexed [23]. If the force exceeds the tensile strength of the tendon, especially in a degenerative state, a rupture can occur, similarly to rupture mechanisms in sprinting and basketball [21,24].

Outcomes following Achilles tendon ruptures have been extensively evaluated in the elite athletic population, including professional athletes participating in organizations such as the National Basketball Association (NBA) [25,26,27], National Football League (NFL) [28,29,30], and Major League Baseball (MLB) [31]. Overall, these injuries are associated with concerning rates of return to play (RTP) together with deterioration in post-injury player performance metrics [21,24,25,32,33,34]. However, little is known regarding the outcomes following Achilles tendon ruptures in elite ice hockey players participating in the National Hockey League (NHL). While no studies have been conducted in NHL athletes regarding outcomes following Achilles tendon ruptures, there are several studies investigating outcomes following other musculoskeletal injuries in NHL athletes [3,35,36,37,38,39,40,41,42,43]. Swindell et al. reported on return to play and performance following episodes of shoulder instability and found that, although the RTP rate was 98.5%, player performance decreased in terms of mean time on ice per game (TOI/G) as well as shooting percentage [39]. Longstaffe et al. found similar results when investigating anterior cruciate ligament (ACL) injuries in NHL players, with a mean RTP rate of 95% but with a decrease in points and goals per game per season upon return [40]. These findings suggest that while NHL players frequently return to sport following various injuries, there is often a decline in key performance metrics, such as time on ice, shooting percentage, and points per game, indicating that the ability to return to play does not necessarily equate to a return to pre-injury performance levels

Thus, the purpose of this study was to assess Achilles tendon ruptures in elite athletes participating in the NHL. We sought to evaluate the incidence, mechanism of injury, treatment strategies, return to play (RTP) rates, and post-injury performance metrics. Our hypothesis is that NHL players will have a high RTP rate, as was the case in NHL players with other injuries, however, they will have diminished post-injury performance metrics due to the debilitating effect of Achilles tendon injuries, as seen in other sports.

## 2. Materials and Methods

### 2.1. Patient Identification

A retrospective review of all NHL players who sustained a partial or complete rupture of the Achilles tendon from 2008 to 2024 was performed. All Achilles tendon ruptures from the 2008–2024 NHL seasons were obtained through a review of injury transactions, injury reports, and injured reserve (IR) placements. Data were obtained from four separate, cohesive, publicly available databases (Hockey-Reference.com, ProSportsTransactions.com, CBSSports.com, and ESPN.com), which have been utilized as valid sources of injury-related data for several previous professional sports epidemiological studies [44,45,46,47,48,49,50,51]. The data were collected by the authors (JJB, RP, NPM, and SK), each with an in-depth knowledge and understanding of the NHL. Each of the four databases were accessed for data and cross-referenced for accuracy. Verification of Achilles tendon rupture injury was conducted using historical reports from the following news sites: NHL.com and Hockey-Reference.com, the latter of which gets its information from Sportradar US, the official statistics partner of the NHL.

The primary inclusion criterion was professional hockey players in the NHL who sustained a partial or complete Achilles rupture from the 2008 to 2024 season. The exclusion criteria were Achilles tendon injuries that did not result in rupture, concomitant injury, and reports prior to 2008. Online statistics prior to 2008 suffered from inconsistent reporting. By 2008, several key sources used in this study, such as Hockey-Reference.com, ProSportsTransactions.com, and CBSSports.com, had established comprehensive and continuous injury and player transaction tracking, supported by reliable third-party data providers like Sportradar US.

### 2.2. Data Extraction

A sports database was created where the following data were extracted: player demographics, number of games played prior to injury, player position, mechanism of injury, if surgical intervention was warranted, return to play data, and post-injury performance data (Table 1). Return to play data included time to return to play and number of NHL matches missed and was calculated as the difference between the date of injury to date of return to NHL play. Post-injury performance data included games played, goals, assists, points, plus/minus, penalty minutes, power play goals, power play points, short-handed goals, short-handed points, time on ice per game, game-winning goals, overtime goals, shots, shooting percentage, and faceoff win percentage (Table 2). Mean performance metrics per season played were obtained pre- and post-injury. This was calculated as the mean value for the performance metric divided by the number of seasons played. Additionally, performance metrics for the season directly pre-injury and the season directly post-injury were obtained, as well as cumulative statistics for all years pre-injury and post-injury (Table 3).

### 2.3. Statistical Analysis

All analyses were performed using SPSS Statistics 28.0.1.1 (IBM, Armonk, NY, USA). Descriptive statistics were calculated with categorical data reported as counts with percentages and continuous data as mean ± standard deviation. A Wilcoxon signed rank test was employed to compare pre-injury and post-injury performance metrics. A *p*-value of <0.05 was determined to be statistically significant.

## 3. Results

### 3.1. Demographic Data and Player Characteristics

Demographic data and player characteristics can be found in Table 1. There was a total of 15 NHL players included in the study who sustained an Achilles tendon rupture between 2008 and 2024, with a prevalence rate of 0.125 injuries per 10,000 athletic exposures. The mean age was 27.8 ± 3.87 years, and the mean body mass index was 26.4 ± 1.51. Prior to injury, athletes played a mean number of games of 462.2 ± 228.9 and a total of 328.9 ± 284.9 games post-injury (*p* < 0.005). In total, 7 athletes played Center (46.7%), 1 played Right Wing (6.7%), 5 were Defenseman (33.0%), 1 played Goalie (6.7%), and no players were Left Wing players (0.0%). The cumulative career of each of the included players included a mean total of 718.9 ± 312 total career games with 13,492.3 ± 6413.7 min on ice for a ratio of 19.6 ± 7.4 time on ice per game for their careers.

### 3.2. Injury Mechanism and Treatment Strategies

Overall, 11 players (73.3%) sustained a non-contact injury, and 4 players (26.7%) sustained a direct laceration to the Achilles tendon. Of the 15 Achilles tendon ruptures, 9 (60.0%) occurred during off-season training and 6 (40.0%) occurred during in-season play. Fourteen athletes (93.3%) underwent operative repair. In total, there were no re-ruptures after operative repair (0.0%).

### 3.3. Performance Metrics Prior to Injury and Post-Injury

**Figure 1:**
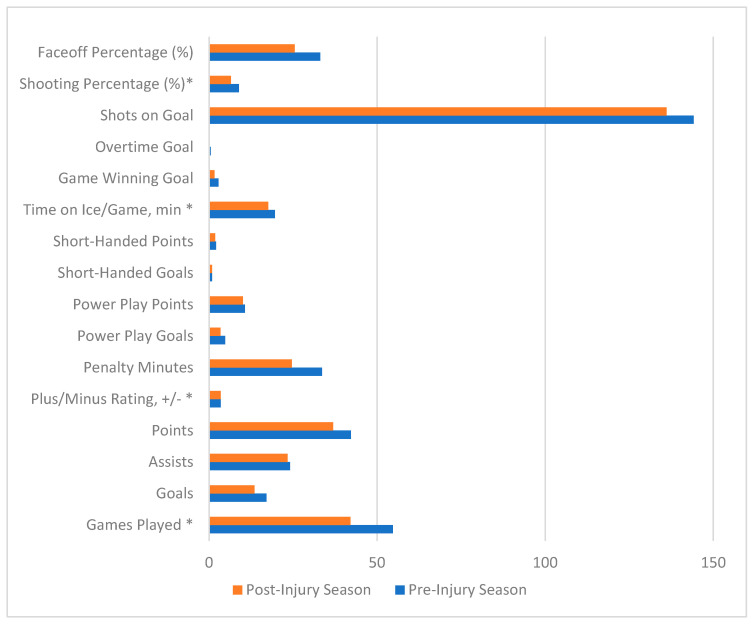
Performance metrics of NHL players with Achilles tendon rupture directly one season pre- and post-injury. * Represents *p*-values < 0.05.

The return to play and performance metrics for the season just prior to injury and just post-injury are highlighted in Table 2 and Figure 1. In total, 14 players (93.3%) returned to play following Achilles tendon ruptures. The mean number of games missed during post-injury rehabilitation was 45.7 ± 21.2 games. The mean number of months from injury to RTP was 4.6 ± 2.0 months. There was a statistically significant reduction in the mean number of games played per season from 54.7 games played pre-injury to 42.1 games played post-injury (*p* = 0.0291). There was a statistically significant reduction in plus/minus rating from pre-injury to post-injury (*p* = 0.0369). There was a statistically significant decrease in time on ice per game (TOI/G), from 19.6 min pre-injury to 17.6 min post-injury (*p* = 0.0244). There was a statistically significant decrease in shooting percentage, which reduced from 8.9% pre-injury to 6.5% post-injury (*p* = 0.0069). There were no statistically significant differences between pre-injury and post-injury goals scored, assists, points, penalty minutes, power play goals, power play points, short-handed goals, short-handed points, game-winning goals, overtime goals, shots on goal, or faceoff percentages pre- and post-injury.

**Figure 2:**
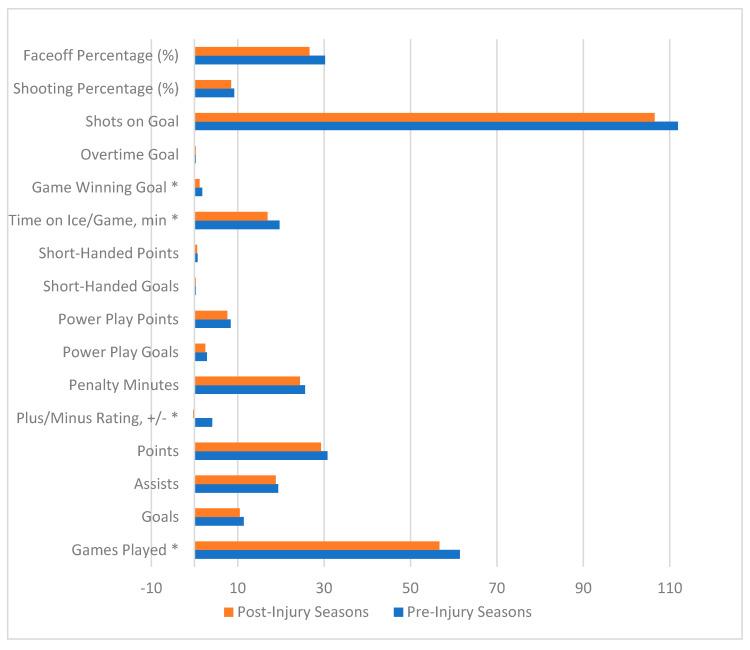
Performance metrics of NHL players with Achilles tendon rupture in all pre-injury seasons and post-injury seasons. * Represents *p* < 0.05.

Table 3 and Figure 2 highlight the mean performance metrics from all seasons pre-injury and post-injury. There was a statistically significant reduction in the mean number of games played per season, from 61.4 games played to 56.7 games played (*p* = 0.0325). There was a statistically significant reduction in plus/minus rating from 4.1 pre-injury to −0.3 post-injury (*p* = 0.0431). Additionally, there was a significant reduction in game-winning goals (GWG; *p* = 0.0276) and TOI/G (*p* = 0.03). There were no statistically significant differences between penalty minutes, power play goals, power play points, short-handed goals, short-handed points, over time goals, shots on goal, shooting percentage, or faceoff percentage from pre-injury to post-injury.

## 4. Discussion

The most important finding of this current study was that Achilles tendon tears are an uncommon injury in NHL players, with a prevalence rate of 0.125 injuries per 10,000 athletic exposures. A high RTP rate of 93.3% was observed in this cohort. However, there was a deterioration in post-injury performance metrics, including games played per season, plus/minus rating, and time on ice per game post-injury, highlighting the potential devastating sequelae of Achilles tendon tears in elite NHL athletes. This confirmed our initial hypothesis of high RTP rates with worse post-injury performance metrics compared to pre-injury.

The Achilles tendon is the strongest tendon in the body and is primarily composed of type I collagen fibers organized in parallel into fascicles, bound by interfascicular matrix and loose connective tissue [17]. Healthy tendons have slow metabolism and are hypocellular compared to other tissues [52,53]. Collagen type I is the key protein in tendons, and makes up roughly 60–85% of the dry weight, with the remaining consisting of proteoglycans, glycosaminoglycans, glycoproteins, and other collagen subtypes, most notably types III, V, and XII [15]. The tenocytes uniformly align along the length of the collagen fibrils and control the metabolism of the tendon. They stretch along the collagen fibrils upon exposure to tensile load, which is a signal for collagen production [17]. Therefore, ECM turnover of the tendon is influenced by physical activity, as well as the blood flow, oxygen demand, the volume of collagen synthesis, and the antagonistic of matrix metalloproteinases (MMPs) during mechanical loading. During inactivity, collagen and ECM turnover drops significantly [16]. The tendon is surrounded by the peritenon, which is a dual-layered connective tissue sheath made up of the epitenon and the paratenon. This sheath acts as a synovial membrane to a joint and allows the tendon to glide effortlessly during motion. The simplest unit of a tendon is the tendon fascicle, which is enclosed by the endotenon that is rich in cells and contributes to the tendon’s blood supply [54]. The tendon is well vascularized at the outer sheaths; however, the core of the tendon has a low cellularity and vascularity [55,56], particularly at the midsection.

Despite the Achilles tendon being the thickest and strongest tendon in the human body, it remains particularly vulnerable to injury, especially in the context of high-intensity sports such as ice hockey. NHL players are uniquely vulnerable to Achilles tendon injuries due to two distinct mechanisms: degenerative overuse injuries and traumatic lacerations. Similar to other athletes, NHL players experience Achilles tendinosis from repetitive eccentric loading during skating, which places significant stress on the tendon. The biomechanics of skating involve powerful plantarflexion and dorsiflexion during rapid acceleration, sudden stops, and direction changes, creating microtrauma over time. This repetitive strain leads to pathophysiological changes, including collagen disorganization, neovascularization, and ECM degradation, all of which weaken the tendon and predispose it to rupture [22]. These changes are characterized by the loss of structural organization of collagen and alterations in the ECM protein content. In the initial phase of tendon damage, collagen type III is produced [19], which acts as a rapid patch to protect the area of damage. Type III collagen, unlike the parallel organization of type I, is laid down haphazardly, and contributes to inferior biomechanical strength and irregular alignment [18]. In normal healing, the type I collagen will eventually replace the type III collagen, and resume the linear organization of collagen fibers [57]. In chronic overuse, as is the case in many athletes, this repair mechanism is impaired, and collagen type III accumulates [58]. During tendinosis, athletes are at particular risk of further injury due to the weaker tensile strength of collagen type III and the reduced ability to bear the shear forces [10]. In ice hockey players, the rigid confines of the skate boot exacerbate stress on the tendon by limiting natural ankle mobility, concentrating forces on the tendon during propulsion. These forces acting on a weakened tendon can once again predispose it to rupture [18]. Compounding this is the unique risk of direct trauma from contact with another player’s skate blade, which can cause partial or complete laceration of the tendon. Unlike tendinosis, these injuries are acute and result from the high velocity and sharpness of the skate, often during collisions or pileups [59]. A lacerated Achilles tendon disrupts the integrity of the muscle–tendon unit, impairing force transmission from the calf to the foot and causing immediate loss of plantarflexion strength.

This current study highlighted the significant impact of Achilles tendon injuries on the ability of NHL players to return to play and maintain consistent participation. An encouraging 93.3% of players successfully returned to play following injury and operative repair, which aligns with return-to-play rates observed in other professional sports, such as the NBA and MLB [21,27,33]. However, despite the high rates of return to play, a reduction in participation post-injury was evident. Players missed a mean number of 45.7 games during their recovery, and the number of games played decreased significantly both in the season immediately following the injury (from 54.7 to 42.1 games, *p* = 0.0291) and across all post-injury seasons (from 61.4 to 56.7 games per season, *p* = 0.0325). This trend underscores a consistent decline in game participation, likely influenced by a combination of physical limitations, cautious workload management by coaches, and potential psychological effects, such as fear of re-injury [60]. The finding of reduced participation following Achilles tendon tears is consistent with other studies in elite athletes in other major sports leagues. Trofa et al. found that among NBA and MLB athletes who successfully returned to play following Achilles tendon tears, the mean game participation was 75.4% and 81.9% of the total games played the season before injury and at 1 and 2 years post-operatively [21]. Chauhan et al. reported a RTP rate of 80% with reduced offensive performance metrics post-injury [27]. Saltzmann et al. reported a RTP rate of 62% in MLB players who underwent surgical repairs of Achilles tendon ruptures and found that ruptures in the power-generating legs resulted in significantly less plate appearances and increased at-bats with strike outs [31]. This highlights the long-term impact of these injuries, even in elite athletes with access to advanced rehabilitation and surgical techniques.

In addition to decreased participation, the findings of this study revealed a significant deterioration in performance metrics among NHL players following Achilles tendon injuries. The mean plus/minus rating, an important measure of a player’s on-ice effectiveness, declined from a positive score of 4.1 pre-injury to a negative score of −0.3 post-injury (*p* = 0.0431). This reduction suggests a measurable decline in players’ ability to contribute to team success during even-strength play. Other performance indicators, such as game-winning goals and time on ice per game, also demonstrated statistically significant declines (*p* = 0.0276 and *p* = 0.0300, respectively), reflecting a diminished role in clutch situations and overall ice time. While the shooting percentage dropped significantly (from 8.9% to 6.5%, *p* = 0.0069), there were no statistically significant changes in offensive metrics, such as goals, assists, or points, indicating that while players continued to generate scoring opportunities, their efficiency in converting those chances was compromised. Interestingly, certain metrics, such as power play performance, short-handed contributions, and faceoff percentages, remained relatively stable, suggesting that Achilles tendon injuries may not uniformly affect all aspects of performance in elite ice hockey athletes. The reduction in player performance metrics following Achilles tendon injuries is consistent with previous studies in other major sports leagues, which have shown a decline in specific performance metrics post-injury despite an overall return to competitive play [21,24,27,30,31,33]. Together, these data emphasize that while NHL players can return to the ice after Achilles tendon injuries, their ability to perform at pre-injury levels may be persistently affected, particularly in areas requiring explosiveness and endurance.

This current study found that Achilles tendon tears in NHL players predominantly occurred during off-season training (60%) and were often non-contact in nature (73.3%), with only one player requiring surgical intervention. Off-season injuries, typically arising during intensified training regimens, highlight a critical area for preventive efforts. These periods of off-ice training, while vital for improving strength and conditioning, also present risks due to increased workloads on the Achilles tendon that is not yet conditioned for the intensity of in-season play. As a result, diligent pre-operative targeted calf exercises may enhance the tendon’s resilience, while flexibility training reduces the risk of tendon strains and tears [61]. In terms of reducing laceration injuries, protective equipment, such as Kevlar socks, specialized padding, and Achilles tendon guards, may also help prevent tendon lacerations resulting from high-speed impacts and sharp skates [62]. These features aim to absorb shock and reduce the risk of direct trauma to the tendon, particularly in situations where players are exposed to sudden impacts or awkward movements.

This study is limited by numerous biases and confounders. One limitation of this study is the relatively small sample size, which reflects the rarity of Achilles tendon ruptures in professional ice hockey players. While this limits the statistical power to detect small differences in outcomes, it is important to note that the population under investigation is inherently limited in size due to the elite and highly selective nature of the cohort. Furthermore, the rarity of this injury type, even within high-contact sports, has similarly constrained sample sizes in prior studies of professional athletes across the NFL, NBA, and MLB [21,24,27,29,30]. Despite this limitation, our study included all confirmed cases over a 16-season span using verified and reproducible methodology, allowing for meaningful clinical insights into injury patterns, return-to-play timelines, and post-injury performance.

Characterization of RTP timelines and performance outcomes can provide evidence-based guidance for rehabilitation planning, expectation management, and player utilization by team physicians and coaching staff. Furthermore, no data regarding the severity of the Achilles tendon tear nor the precise post-injury rehabilitation protocol were described. Additionally, no control cohort was included.

Future research should focus on large prospective studies that would explore the long-term outcomes of Achilles tendon ruptures in elite athletes. Comparisons of post-injury rehabilitation protocols and their effects on the number of games missed and post-injury performance metrics would broaden the evidence base so that teams can make more accurate and effective decisions.

## 5. Conclusions

This study found that Achilles tendon ruptures are an uncommon injury in NHL players, with a prevalence rate of 0.125 injuries per 10,000 athletic exposures. A high RTP rate of 93.3% was observed in this cohort. However, there was a deterioration in post-injury performance metrics, including games played per season, plus/minus rating, and time on ice per game post-injury, highlighting the potential devastating sequelae of Achilles tendon ruptures in elite NHL athletes.

## Figures and Tables

**Table 1:** Demographic characteristics of NHL players with Achilles tendon injury.

Age (SD)	27.8 (3.87)
Body Mass Index (SD)	26.4 (1.51)
Career GP (SD)	718.9 (312.0)
Career TOI, min	13,492.3 (6413.7)
TOI/GP	19.6 (7.4)
Total Games Pre-Injury	486.2 (228.9)
Total Games Post-Injury	328.9 (284.9)
Player Position	
Center (%)	7 (46.7%)
Left Wing (%)	0 (0.0%)
Right Wing (%)	1 (6.7%)
Defenseman (%)	5 (33%)
Goalie (%)	1 (6.7%)
Mechanism of Injury	
Non-Contact Injury (%)	11 (73.3%)
Laceration (%)	4 (26.7%)
Timing of Injury	
Off-Season (%)	9 (60.0%)
In-Season (%)	6 (40.0%)
Injury On- vs. Off-Ice	
On-Ice (%)	9 (60.0%)
Off-Ice (%)	6 (40.0%)
Treatment	
Surgical (%)	14 (93.3%)
Non-Surgical (%)	1 (6.7%)

**Table 2:** Performance metrics of NHL players with Achilles tendon rupture, pre- and post-injury seasons.

	Pre-Injury Season	Post-Injury Season	*p*
Return to Play (%)	14 (93.3%)		
Return to Play (Months, SD)	4.6 (2.0)		
Games Missed (SD)	45.7 (21.2)		
Games Played (SD)	54.7 (15.2)	42.1 (15.1)	0.0291
Goals (SD)	17.1 (24.5)	13.5 (24.2)	0.6886
Assists	24.1 (29.3)	23.4 (24.2)	0.9436
Points	42.2 (53.6)	36.9 (55.6)	0.7923
Plus/Minus Rating, +/− (SD)	3.5 (7.1)	3.5 (7.2)	0.0369
Penalty Minutes (PIM)	33.6 (37.4)	24.6 (38.1)	0.5192
Power Play Goals (PPG)	4.8 (5.7)	3.4 (5.7)	0.5067
Power Play Points (PPP)	10.7 (11.5)	10.1 (13.1)	0.8949
Short-Handed Goals (SHG)	0.9 (2.6)	0.9 (2.7)	1.0000
Short-Handed Points (SHP)	2.1 (5.8)	1.8 (5.8)	0.8884
Time on Ice/Game, min (TOI/G)	19.6 (2.2)	17.6 (2.4)	0.0244
Game-Winning Goal (GWG)	2.8 (2.8)	1.6 (2.1)	0.1949
Overtime Goal (OTG)	0.5 (0.8)	0.2 (0.6)	0.2551
Shots on Goal (S)	144.2 (179.7)	136.1 (189.2)	0.9052
Shooting Percentage (S%)	8.9 (2.1)	6.5 (2.4)	0.0069
Faceoff Percentage (FO%)	33.1 (26.0)	25.5 (26.8)	0.4371

**Table 3:** Performance metrics of NHL players with Achilles tendon rupture, pre-injury seasons and post-injury seasons combined.

	Pre-Injury Total Seasons	Post-Injury Total Seasons	*p*
Games Played	61.4 (5.2)	56.7 (6.3)	0.0325
Goals	11.4 (7.5)	10.5 (8.5)	0.7607
Assists	19.4 (9.4)	18.8 (13.5)	0.8887
Points	30.8 (6.1)	29.3 (2.1)	0.3755
Plus/Minus Rating, +/− (SD)	4.1 (5.0)	−0.3 (6.3)	0.0431
Penalty Minutes (PIM)	25.6 (10.5)	24.4 (11.2)	0.7643
Power Play Goals (PPG)	2.9 (2.3)	2.5 (2.4)	0.8448
Power Play Points (PPP)	8.4 (6.1)	7.6 (7.3)	0.7471
Short-Handed Goals (SHG)	0.3 (0.4)	0.3 (0.5)	1.0000
Short-Handed Points (SHP)	0.7 (0.6)	0.6 (1.0)	0.7423
Time on Ice/Goals (TOI/G)	19.7 (3.9)	16.9 (2.7)	0.0300
Game-Winning Goal (GWG)	1.8 (0.8)	1.2 (0.6)	0.0276
Overtime Goal (OTG)	0.3 (0.3)	0.3 (0.4)	1.0000
Shots on Goal (S)	111.9 (59.1)	106.5 (69.2)	0.8199
Shooting Percentage (S%)	9.2 (3.9)	8.5 (4.4)	0.6483
Faceoff Percentage (FO%)	30.2 (24.2)	26.6 (26.5)	0.7184

## Data Availability

The data that support the findings of this study are publicly available from the following online sources: Hockey-Reference.com, ProSportsTransactions.com, CBSSports.com, and ESPN.com. All data were accessed in accordance with the respective websites’ terms of use. No new data were generated by the authors that are not already publicly available.

## Abbreviations

The following abbreviations are used in this manuscript:

NHL	National Hockey League
RTP	Return to Play
NBA	National Basketball Association
NFL	National Football League
MLB	Major League Baseball
GWG	Game-Winning Goal
TOI	Time on Ice
TOI/G	Time on Ice Per Game
